# Caveat Medicus: Clinician experiences in publishing reports of serious oncology-associated adverse drug reactions

**DOI:** 10.1371/journal.pone.0219521

**Published:** 2019-07-31

**Authors:** Charles L. Bennett, Benjamin Schooley, Matthew A. Taylor, Bartlett J. Witherspoon, Ashley Godwin, Jayanth Vemula, Henry C. Ausdenmoore, Oliver Sartor, Y. Tony Yang, James O. Armitage, William J. Hrushesky, John Restaino, Henrik S. Thomsen, Paul R. Yarnold, Terence Young, Kevin B. Knopf, Brian Chen

**Affiliations:** 1 The University of South Carolina College of Pharmacy, Columbia, South Carolina, United States of America; 2 College of Engineering and Computing, University of South Carolina, Columbia, South Carolina, United States of America; 3 University of South Carolina School of Medicine, Columbia, South Carolina, United States of America; 4 Medical University of the University of South Carolina, Charleston, South Carolina, United States of America; 5 Tulane University School of Medicine, New Orleans, Los Angeles, United States of America; 6 George Washington University, Washington, Washington, D.C., United States of America; 7 University of Nebraska Medical Center, Omaha, Nebraska, United States of America; 8 Arnold School of Public Health, University of South Carolina, Columbia, South Carolina, United States of America; 9 Copenhagen University Hospital, Herlev, Denmark; 10 Drug Safety, Oakville, Ontario, Canada; 11 Alameda Health System, Oakland, California, United States of America; Hospital JP Garrahan, ARGENTINA

## Abstract

Oncology-associated adverse drug/device reactions can be fatal. Some clinicians who treat single patients with severe oncology-associated toxicities have researched case series and published this information. We investigated motivations and experiences of select individuals leading such efforts. Clinicians treating individual patients who developed oncology-associated serious adverse drug events were asked to participate. Inclusion criteria included having index patient information, reporting case series, and being collaborative with investigators from two National Institutes of Health funded pharmacovigilance networks. Thirty-minute interviews addressed investigational motivation, feedback from pharmaceutical manufacturers, FDA personnel, and academic leadership, and recommendations for improving pharmacovigilance. Responses were analyzed using constant comparative methods of qualitative analysis. Overall, 18 clinicians met inclusion criteria and 14 interviewees are included. Primary motivations were scientific curiosity, expressed by six clinicians. A less common theme was public health related (three clinicians). Six clinicians received feedback characterized as supportive from academic leaders, while four clinicians received feedback characterized as negative. Three clinicians reported that following the case series publication they were invited to speak at academic institutions worldwide. Responses from pharmaceutical manufacturers were characterized as negative by 12 clinicians. One clinician’s wife called the post-reporting time the “Maalox month,” while another clinician reported that the manufacturer collaboratively offered to identify additional cases of the toxicity. Responses from FDA employees were characterized as collaborative for two clinicians, neutral for five clinicians, unresponsive for negative by six clinicians. Three clinicians endorsed developing improved reporting mechanisms for individual physicians, while 11 clinicians endorsed safety activities that should be undertaken by persons other than a motivated clinician who personally treats a patient with a severe adverse drug/device reaction. Our study provides some of the first reports of clinician motivations and experiences with reporting serious or potentially fatal oncology-associated adverse drug or device reactions. Overall, it appears that negative feedback from pharmaceutical manufacturers and mixed feedback from the academic community and/or the FDA were reported. Big data, registries, Data Safety Monitoring Boards, and pharmacogenetic studies may facilitate improved pharmacovigilance efforts for oncology-associated adverse drug reactions. These initiatives overcome concerns related to complacency, indifference, ignorance, and system-level problems as barriers to documenting and reporting adverse drug events- barriers that have been previously reported for clinician reporting of serious adverse drug reactions.

## Introduction

Oncology-associated adverse drug/device reactions are serious medical toxicities. These toxicities encompass three areas. Oncology drugs are the most toxic pharmaceutical class [1,2]. Oncology patients are ill, with comorbid illnesses, extensive cancer, and undergoing toxic treatments. When adverse reactions occur that represent severe oncology-drug induced gastrointestinal toxicity, arterial or venous thromboembolism, cardiac arrhythmias, opportunistic infections, or hemorrhage, these events are frequently attributed to the cancer, comorbid illness, or the “cost of doing business.” [3–6] A second toxicity type relates to oncology-drug induced growth or dissemination of existing cancers- as with erythropoiesis stimulating agents or morcellator procedures.[7,8] A third type relates to new cancer development as postulated with natalizumab associated melanoma or protein pump inhibitors associated gastric malignancies.[9,10] Two National Institutes Health R01 funded pharmacovigilance programs (the Southern Network on Adverse Reactions (SONAR) and the Research on Adverse Drug Events and Reports (RADAR), projects), conducted beginning in 1998, have collaborated with clinicians to report > 50 serious adverse drug/device reactions from the oncology setting.[3,6] These co-investigators are aware of efforts of academic collaborators who also reported similar serious oncology-associated adverse drug reactions. SONAR and RADAR co-investigators and colleagues have personally treated oncology patients who developed several life-threatening adverse drug reactions, evaluated these patients, and published on the cause and cases of these toxicities. These investigations were frequently followed by label warnings and/or convening of Food and Drug Administration (FDA) Advisory Committee reviews of safety findings. Clinicians who take “extra-steps” of evaluating serious adverse drug reactions and reporting this in medical publications may be subject to repercussions or alternatively, career enhancements. Our purpose is to shed light on motivations and experiences of clinicians who worked with investigator leaders of two National Institutes supported pharmacovigilance networks and who also personally evaluated and reported unrecognized serious adverse drug reactions.

## Materials and methods

Our study is a qualitative analysis of experiences who collaborate with leaders of two NIH funded pharmaceutical safety grants and subsequently published case series of serious oncology-associated (adverse drug reactions). The ADRs were initially seen as an individual case by the interviewed clinician. We used “The Criteria for reporting qualitative research (COREQ): a 32-item checklist for interviews and focus groups” to guide the design, analysis, and reporting of our study.[11] As recommended by the COREQ guidelines, the qualitative researchers in this study varied in credentials, training, occupation, and training. The principal investigator was a qualitative analyst PhD researcher. A senior co-investigator/interviewer was a MD PhD health services researcher/policy endowed chair professor who had previously published physicians reporting of health care fraud, and another co-investigator was a JD PhD health policy tenured professor who had conducted qualitative and quantitative research, including one manuscript on physicians reporting of health care fraud. The theoretical framework was based on an exploratory approach to support hypothesis generation- relevant considerations since there are no published data about clinician experiences with identifying and reporting case series of serious adverse-drug reactions. The analytic focus was based on quantitative analysis and systematic organization of data into a structured format.

**Fig 1:**
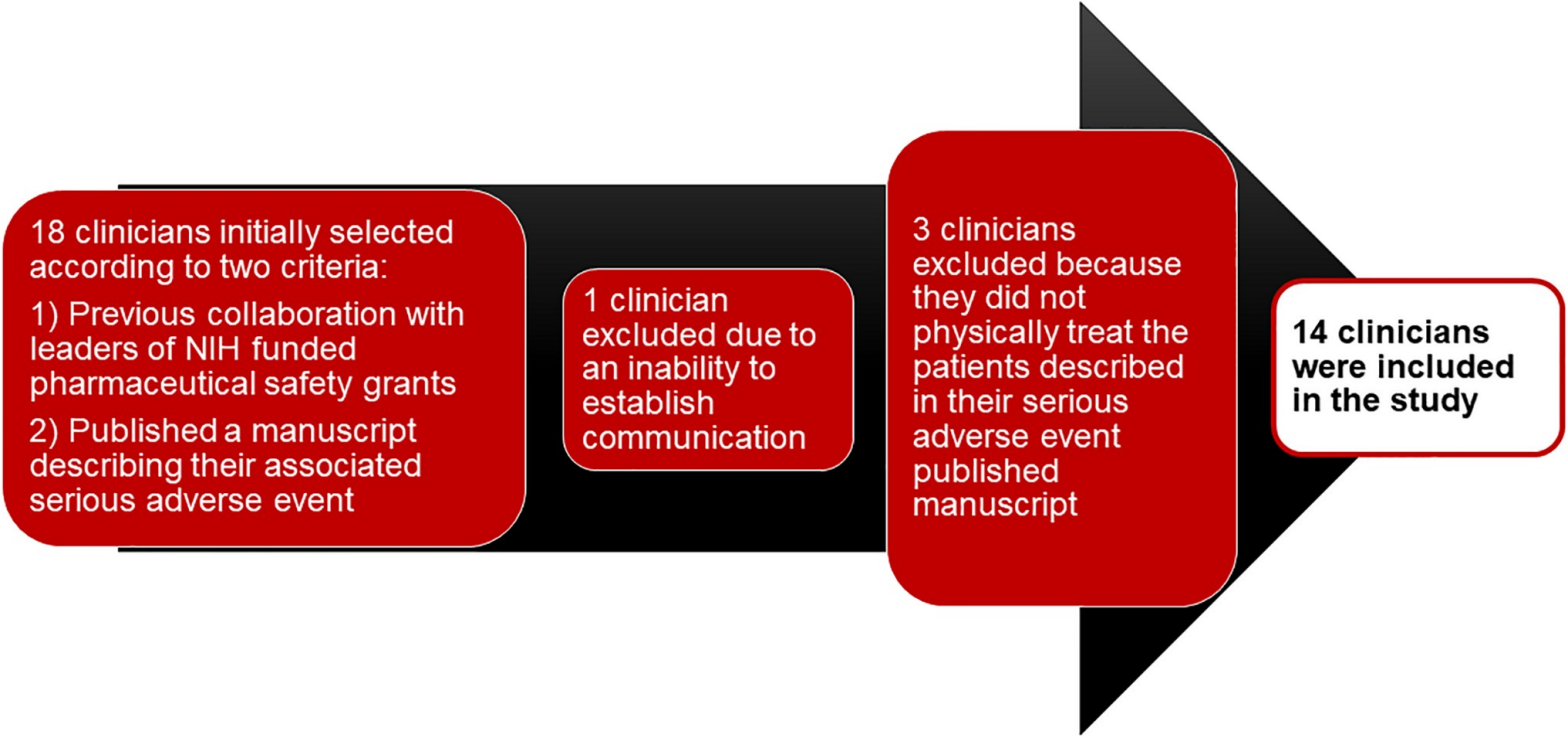
Flow chart for study inclusion criteria.

No repeat interviews were conducted. After 14 interviews were conducted and the responses analyzed, the Principal Investigator, the co-Principal Investigator, and the study team jointly determined that saturation had occurred. Transcripts were not returned to the interviewees, but were reviewed by the co-Principal Investigator. Data coding and transcription were done by four research assistants, with two research assistants independently coding and transcribing each interview. The co-Principal Investigator provided to the research assistants the agreed upon coding terms that guided transcript analysis. Themes were derived in advance, based in part on personal experiences of the co-Principal Investigator treating persons with serious oncology-associated adverse drug reactions and subsequently reporting this information in the medical literature. Since the interview transcript size was small (about five pages per interview), no software analysis package was used. Participants reviewed the draft manuscript (prior to submission) and were asked to provide relevant feedback, particularly if each interviewee did not express concerns about linking of responses to individual interviewees. Quotations were presented to illustrate each of the selected themes. Participant numbers are included in the quotations. The data and the findings exhibited strong consistency, as assessed by the study investigators. The major themes are presented in each table and in the corresponding text. Minor themes are also highlighted in the tables and the text. Overall, the research team, study design, and analysis/findings were consistent with the 32 consolidated criteria for reporting qualitative studies (COREQ).[11] The details of the process are shown in a flow chart in Fig 1.

The qualitative approach was chosen as it is a rigorous approach for investigating personal motivations, reflections, and outcomes among a small group of persons who have a common experience.[12] Qualitative methods are also considered appropriate for studies that provider exploratory research and hypothesis generation. These are appropriate considerations as there are no prior research publications that report on clinician motivations for publishing serious adverse drug reaction information. The study received approval from the University of South Carolina Institutional Review Board. All participants provided verbal consent.

The operational definition of serious adverse drug reactions were events attributed by the reporting clinician as causing death or potentially fatal organ toxicity. Publication dates are redacted to ensure clinician anonymity. Most reports were followed by boxed warnings and/or FDA Advisory Committees. The study interviewer was an MD PhD MPP interviewer whose research focused on pharmaceutical safety research and impacts of this work on academic clinicians who report serious adverse drug reactions. This interviewer was trained by an experienced qualitative research journalism professor who also conducted the first interview as part of the training session. The interview guide was developed by the experienced qualitative researcher. (S1 Interview Guide) The guide underwent field review by three additional PhD health services researchers with expertise in drug safety. The guide contained a list of key thematic areas to be probed for clarification and additional detail for each of four pre-selected topic areas- interviewee views of responses by employees of the pharmaceutical manufacturers, employees of the FDA, and clinicians in academic medical centers and recommendations that the interviewee considered as relevant for future pharmacovigilance efforts. All interviews were recorded and transcribed. No interviewee requested a copy of the interview guide prior to the interview. The interviewer also kept written notes. The interview began by obtaining verbal informed consent, followed by demographic and professional information on the interviewee. Next, we elicited information on the nature of the work that led to identification of the index case of the serious adverse drug reaction and to their follow-on work to establish a case series. Interviews then proceeded chronologically through the following topics: the interviewees personal assessment of responses to the study findings by employees of the relevant pharmaceutical manufacturer, safety personnel of the FDA, clinicians in academia at either the home institution of the interviewee or an academic institution where the interviewee had applied for a possible academic position, and recommendations by the interviewee that might be considered when future studies evaluate potential serious adverse drug reactions. Interviewees were asked to verify that they had not participated in any “whistle blower” investigation and had not received any income for publishing their work.

We analyzed interview transcripts using standard coding techniques and the constant comparative methods of qualitative analysis, based on methods reported previously.[13,14,15] The two senior investigators independently analyzed the interviews and developed separate coding schemes for organization the data. Coding schemes were then compared, discussed, and reconciled to product a final coding structure which consisted for six broad themes covering 20 specific codes. One investigator (CLB) then used the final coding scheme to code all of the transcripts and interview notes, with input from each of the four transcribers. The transcripts and interview notes were coded using manual counts for each of the 20 specific codes. The analysis focused on organizing and describing themes, including distinctions among clinicians based on their academic training (clinician/PhD versus clinician) and level of training (fellow or student versus assistant or associate professor).

## Results

We identified 18 eligible persons for interview. Three interviewed persons were excluded as they did not have first-hand information on index cases. One clinician who was working at a pharmaceutical corporation did not return telephone calls. We conducted semi-structured 30-minute telephone interviews at the workplace with persons who we identified as clinicians who were collaborators with a key investigator of SONAR and RADAR projects or who collaborated closely with a senior RADAR/SONAR co-investigator, had evaluated a case of a serious oncology-associated adverse drug reaction, personally conducted research to identify cases of persons with this toxicity, and then published the findings in peer-reviewed medical journals or, in one case, presented findings to FDA personnel. The study sample was felt to be exhaustive of all individuals who met the entry criteria. All but one of the 15 eligible participants were interviewed- the lone exception had left academic medicine and could not be contacted. The contacted clinician received an email querying if there would be interest, then a phone call from the study co-Principal Investigator confirming interest in participation, and then was emailed a study description that included a consent form. A telephone interview was conducted with the study participants with a research assistant taking notes and taking the conversation and the co-Principal Investigator and the interviewee speaking for about 30 minutes. Three potential participants were not involved in clinical medicine. The characteristics of the interviewed persons are outlined in [Table 1]. The interview guide was developed by a journalism PhD collaborator with extensive expertise in qualitative research and telephone-based interview studies. Interviews lasted a median of 35.5 minutes (interquartile range, 30 to 40 minutes) addressed clinician motivation and experiences. All of the 14 included transcripts were found to have > 98% concordance.

**Table 1:** Table of adverse drug reactions.

Training (# Patients with Toxicity) (# Study Sites)	Estimated Rate	FDA Meeting Convened	Disseminated in High-Impact Factor Medical Journal*	Boxed Warning Added to Product Label	Discovery
**Oncologist MD PhD** **(60 Pts)** **(Multi-Site Observational Study)**	1 in 10,000		Yes	Yes	Fatal Brain Infection-Monoclonal Antibody
**Surgeon MD PhD** **(10 Pts)** **(Multi-Site Observational Study)**	1 in 350	Yes		Yes	Device—Metastatic Cancer
**Oncologist MD** **(10 Pts)** **(Phase III NCI Sponsored Clinical Trial)**	1 in 6	Yes	Yes	Yes	Fatal Gastrointestinal Toxicity
**Surgeon DDS** **(20 Pts)** **(Single-Site Observational Study)**	1 in 100	Yes		Yes	Bone Necrosis
**Oncologist MD** **(10 Pts)** **(Single-Site Observational Study)**	1 in 25		Yes		Secondary Cancer
**Surgeon DDS PhD** **(40 Pts)** **(Single-Site Observational Study)**	1 in 100	Yes		Yes	Bone Necrosis
**Oncologist MD** **(110 Pts)** **(Phase III Industry Sponsored Clinical Trial)**	1 in 10	Yes	Yes	Yes	Tumor Progression
**Surgeon MD DMD** **(70 Pts)** **(Single-Site Observational Study)**	1 in 100	Yes		Yes	Bone Necrosis
**Surgeon MD PhD** **(340 Pts)** **(Meta-Analysis)**	1 in 4	Yes	Yes	Yes	Tumor Progression
**Oncologist MD** **(30 Pts)** **(Single Site Observational Study)**	1 in 3		Yes		Arterial Vascular Toxicity
**MD PhD Student** **(10 Pts)** **(Multi-Site Observational Study)**	Not available				New Cancer Development
**Oncologist MD PhD** **(10 Pts)** **(Industry Sponsored Clinical Sites in a Phase III Clinical Trial)**	Not available		Yes		Cardiac Toxicity
**Oncologist MD** **(10 Pts)** **(Single Site Observational Study)**	1 in 5		Yes	Yes	Venous Thromboembolism
**Pharmacist PharmD** **(20 Pts)** **(Single Site NCI Sponsored Clinical Trial Study)**	1 in 5				Venous Thromboembolism

*High impact factor medical journals included the New England Journal of Medicine, the Journal of the American Medical Association, the Annals of Internal Medicine, Blood, the Journal of Clinical Oncology, and the Journal of the National Cancer Institute

Overall, data were included from 12 clinicians who had personally treated a patient who developed a serious and previously unreported serious adverse drug reaction, one clinician whose wife died from a previously unrecognized adverse device reaction, and one MD PhD student whose mentor had treated the index patient. Five clinicians had MD/PhD or DDS/PhD degrees, one clinician was enrolled in an MD/PhD program, and six clinicians had extensive basic and/or clinical science training. The sample included six medical oncologists, one radiation oncologist, one oncology PharmD, five surgeons, and one MD PhD student who worked closely with an oncologist oncology mentor. Studies were initiated following review of a single case (n = 3) or after treating between four and 65 cases with the relevant toxicity (n = 11), or after reviewing clinical trial data involving for 350 and 800 patients in two phase III clinical trials (n = 2). Six reports were single-site case series, two were phase II single-site clinical trial reports, one was a meta-analysis, two were phase III clinical trial reports, and three were observational studies from a small number of academic medical centers.

Investigators included one medical student, three oncology fellows, and 10 clinicians who were assistant/associate professors at academic medical centers when the case series was evaluated. Adverse drug reactions included fatal infections of the central nervous system (n = 1), bone osteonecrosis (n = 3), tumor progression/development (n = 5), arterial or venous thromboembolism (n = 3), cardiac arrythmias (n = 1), or fatal gastrointestinal toxicity (n = 1). Five studies were disseminated in four high-impact factor medical journals (JAMA, Lancet, Annals of Internal Medicine, and New England Journal of Medicine), five studies were disseminated in two high-impact factor hematology-oncology journals (Blood and the Journal of Clinical Oncology), two studies were disseminated in two surgical specialty journals, one study was disseminated in a lower impact-factor oncology journal, and one study was disseminated by the Government Accountability Organization.[8]

Following dissemination of safety findings for eleven and seven studies, respectively, boxed warnings were added to product labels and FDA advisory committees were convened. Related peer-reviewed publications were cited in medical publications a median of 1,114 times (range 290 to 2,500 for 13 reports) or in national newspapers (for 7 reports). The median estimated incidence of the adverse events was 1 in 6 treated patients (range 1 in 3 to 1 in 10,000 (13 reports)). Five described toxicities that were fatal. Following seven adverse drug reaction reports, phase III clinical trials were temporarily discontinued while additional safety evaluations were conducted. For one toxicity, a manufacturer voluntarily discontinued manufacturing the implicated device. Seven study reports were submitted to the FDA for additional review, and 10 investigators submitted related findings to pharmaceutical manufacturers. [Table 1]

### Motivations

All but one of the clinicians intended to publish the findings, generally aiming for publication in a top-tier medical journal or medical/surgical specialty journal. One clinician retained attorneys who filed a device liability lawsuit against a manufacturer and submitted safety data to the FDA, but did not submit safety data to a peer-reviewed journal. Reported motivations coalesced around five non-mutually exclusive themes: scientific curiosity, public health, a desire to inform the broad medical community of a toxicity that had not been reported previously, ethics, and because the toxicity had affected a clinician’s wife.

The most common theme, scientific curiosity, was linked by six clinicians to their training as a clinician scientist. One clinician reported that “the MD side of me identified the toxicity and the PhD side of me facilitated my scientific investigation of the causes of the toxicity”. Another clinician reported that “as a MD PhD investigator, this is the kind of science that I have been trained to do”. Three clinicians reported that training as clinician scientists led to the investigation. Five of six clinicians who reported scientific curiosity as the main motivator were investigators of National Cancer Institute-sponsored or pharmaceutical manufacturer funded investigator-initiated trials.

A less common theme was public health related, with three clinicians reporting that toxicity findings had public health implications that they felt was their responsibility to report. One clinician said “what got me personally was seeing the denials of this information and the callous disregard [of the pharmaceutical manufacturer] for this complication”. A third theme, ethics, was endorsed by two clinicians. One clinician cited the Belmont Report as a motivating factor. One clinician, endorsing personal reasons for toxicity evaluation, stated that “I have to tell you without a question if this problem had not happened to us [the clinician’s spouse died from the toxicity], I would have probably agreed that this was a problem but I wouldn’t have done anything more than that. As many of my colleagues did”. [Table 2]

**Table 2:** Primary motivations for publishing adverse drug reactions.

Primary Motivation	Illustrative Remark
**Scientific Curiosity** **(6 Participants)**	“I did it because it was important. Well, so I did it because it was a curiosity to me scientifically. But, having done an MD/PhD, this is the space that I’m supposed to be in.” (P12) “First of all, I’m a scientist. I’m a doctor, a medical doctor.” (P9) “It was just the love of the game, just pure intellectual curiosity.” (P13) “I think my passion for knowledge and sharing that knowledge, not just with the medical community, but I also really want this information to be disseminated.” (P14) “I take sort of a scientific bend towards thinking about the patients, you know, both in terms of how we treat them clinically and sort of biologically understanding them.” (P15)
**Medical Awareness** **(2 Participants)**	“I think the main thing was that I hope that the profession understands that we have a role in this whole business of medications.” (P5) “I was worried that something bad would happen to people …” (P16)
**Ethics** **(2 Participants)**	“So, in the end, our responsibility as investigators is to each individual patient enrolled in the trial, based on the Belmont Report.” (P4) “What got me going personally was seeing the denials of this information and their callous disregard for this complication.” (P7)
**Public Health** **(3 Participants)**	“I think it was really about wanting transparency around this [toxicity].” (P1) “Well, I think it was public health because a lot of people were taking this drug. So, I thought it was important for them to realize that there was a potential problem.” (P10)
**Personal** **(1 Participant)**	“I have to tell you without a question if this had not happened to us (my wife and I), I would have probably agreed this was a problem, but I wouldn’t have done anything more than that. As many of my colleagues did.” (P2)

### Responses by the academic community

The academic community was reported as being supportive for six clinicians, unfavorable towards four clinicians, and neutral towards two clinicians. Three clinicians reported that following the publishing of their case series, they were invited to speak at academic institutions worldwide. One clinician reported “Well, the chairman of my department said this is really significant…I say, well, mostly, he was right”. In contrast, two clinicians reported that during job interviews, a senior academic interviewer indicated that reporting of the serious adverse drug reactions would not be an academic pursuit that should be continued. One clinician stated “the chairman where I interviewed said you will not make friends with this kind of research”. Two clinicians were not comfortable sharing information about responses that they had received from the medical community. [Table 3]

**Table 3:** Interviewee report of academic community response.

Type of Response	Illustrative Remark
**Positive** **(6 Participants)**	“Well, the chairman of my department said this is really significant… This is going to be something that is going to be really, really significant. I say, mostly, he was right.” (P10) “The university had my back.” (P6) “I speak [by invitation] (on this) all over the world.” (P7) “My institution was very supportive.” (P16)
**Neutral** **(2 Participants)**	“Everyone was kind of happy, but not anything major. They said, ‘OK good publication. Good guy, fine job. What’s next?” (P9) “My chairman saw the paper and [said] ‘you’re taking on [a large pharmaceutical company]…. It’s a billion dollar drug for them…so it’s going to hurt their bottom line.’” (P12)
**Negative** **(4 Participants)**	“The chairman said you will not make friends with this kind of research.” (P1) “I spent the next six years without giving academic talks.” (P13)

### Reponses by the pharmaceutical community

The pharmaceutical community was generally negative towards the clinicians, although two clinicians reported collaborative responses. One clinician said that his wife called the post-reporting time the “Maalox month”–primarily in response to intimidation by a physician employed by a pharmaceutical manufacturer. Another clinician met with the Chief Executive Officer of the relevant pharmaceutical manufacturer who said he wanted to see “in person the [expletive] who had cost us $2 billion”. A third clinician was sued by the pharmaceutical device manufacturer. Safety findings were subsequently central aspect of legal settlements paid by a manufacturer to the Department of Justice of $600 million (two clinicians) and $300 million (two clinicians), although these clinicians did not receive financial rewards. One clinician reported that the manufacturer collaboratively offered to identify additional cases of the relevant toxicity that were in the manufacturer’s safety database. One clinician reported that the relevant manufacturer was fairly negative, while a competitive manufacturer placed this person on an academic advisory panel and sponsored the clinician to meet with safety representatives of the FDA. [Table 4]

**Table 4:** Interviewee report of pharmaceutical company response.

Pharmaceutical Perceptions	Illustrative Remark
**Collaborative** **(2 Participants)**	“I really do commend the company that I worked with because as a ‘fellow’, which is not even a full-fledged physician, I was on the phone with a senior person of the company discussing what I was thinking. They facilitated me getting my hands on a number of cases that they had seen.” (P1) “I think we’re working toward getting more information out there about [this toxicity]…I think they [pharma] would like to do further investigation.” (P13)
**Negative** **(12 Participants)**	“The second communication I got was a threat from their executive vice president that I would be sued for defamation.” (P2) “When our paper came out, they actually tried to discredit me. There was an interview that they gave to a New York Times reporter, where they said [my report] was flawed. And they actually called some of my colleagues and asked them if they knew dirt on me.” (P4) “They [the manufacturers] escaped litigation with disclaimers on television…I was very naive thinking they [pharma] were upstanding…” (P7) “I met with the scientific people from [the company] and we discussed the data. A knock on the door occurred and the CEO walked in, looked at me and said, ‘I just want to look at the [expletive] who lost us 2 billion dollars.’ Then he walked out.” (P9) “They had this white document [about the toxicity] and they didn’t distribute it …The only people who knew about [the toxicity] were a few oncologists who had some concerns about [the toxicity].” (P5) “[The company], particularly, didn’t want hear about negative stuff.” (P10) “I think their goal is more based on financial incentive rather than solely looking out for the benefit of the patient.” (P14) “I think they were unbelievably corrupted when it came to this from an ethical prospective. And it became even worse when we found out that a year prior to the complication another woman was harmed using the same technique, and that she was actually on her deathbed when my wife had the operation. I learned that they had had extensive conversations internally and chose to do nothing to protect patients.” (P2)

### Responses by the FDA

FDA responses were characterized as collaborative for two clinicians, neutral for five clinicians, unresponsive for two clinicians, and negative for six clinicians. One of these instances was described as collaborative and negative. Some persons at the FDA worked with the clinician to facilitate market withdrawal of the implicated device. Other FDA persons were reported as being unresponsive although other clinicians had previously reported device-associated deaths to the FDA. One clinician reported that FDA safety officials were not responsive because he/she “practiced in a foreign country, and that FDA safety officials appeared to be more responsive to United States’ clinicians”. One clinician reported that the response was vigorous and positive- the FDA convened an independent auditing panel to review the safety findings. One clinician who had submitted >60 reports to FDA’s MedWatch database was surprised when no follow-up from FDA personnel occurred. [Table 5]

**Table 5:** Interviewee report of fda employee response.

FDA Responses	Illustrative Remark
**Positive Collaboration** **(2 Participants)**	“Well, I think the FDA responded very responsively and I have to credit [one senior FDA person] for many things. One of the things I credited him/her for is that [he/she] took this seriously.” (P1)
**Neutral Collaboration** **(5 Participants)**	“They know what we’re doing. They smile …” “I remember three years later, when I did a poster [presentation], somebody from the FDA came by and wanted to talk to me about [the toxicity—three years after my report].” (P14)
**Zero/Unresponsive** **(2 Participants)**	“No, I never got a call from the FDA.” (P5)
**Negative Collaboration** **(6 Participants)**	“Unfortunately, I don’t think this had been a major funding priority for the FDA.” (P13) “I think in the end, FDA’s actions and the commissioner’s actions were extremely weak. If a can of soup or food product is causing a mortality risk at a rate of 1 in 350 and the FDA believes that, they would immediately pull that product off the market.” (P2) “I had that one meeting with the FDA… They were more and more focusing on American scientists [which I am not]…” (P9) “Unfortunately, the FDA seems to have bought the Kool-Aid on the drug.” (P7)

### Recommendations for going forward

Five major concepts were endorsed for improving drug safety going forward. Three clinicians endorsed developing improved reporting mechanisms for individual physicians (n = 3). Eleven clinicians endorsed safety activities that should be undertaken by persons other than a motivated clinician who personally treats a patient with a severe adverse drug/device reaction, including supporting independent pharmaceutical watchdog organizations (3 clinicians), retrospective FDA-funded safety analyses (one clinician), novel scientific studies including pharmacogenetics (two clinicians), big data analytics (two clinicians), or improved data safety monitoring boards (one clinician). One of the recommendations supported mandatory safety review of all drugs five years after FDA approval has been granted. One clinician noted that since his/her events were reported, pharmaceutical safety efforts had improved markedly—with widespread utilization of Data Safety Monitoring Boards. Two participants did not endorse any specific recommendation for future safety efforts.

## Discussion

This study of 14 clinicians who have published reports of, or potentially fatal oncology associated adverse drug/device reactions found that responses of the relevant pharmaceutical manufacturer were primarily negative, responses by fellow academic clinicians were generally supportive or neutral, and responses by FDA personnel were supportive in only two instances. The clinical findings were significant, with many reports being published in high-impact medical journals, being described in revised product labels and in “Dear Doctor” letters, and in four instances, being associated with settlements by pharmaceutical manufacturers for > $900 million. In interpreting our findings, several factors should be considered.

The most common motivation was scientific curiosity. This was reported by three of the five PhD clinicians as well as the MD/PhD student who indicated that the physician background facilitated recognition of the toxicity and the PhD background facilitated detailed basic science investigation. In one instance, a clinician reported the toxicity after his wife had developed device-associated metastatic sarcoma (and ultimately died from this toxicity). There are no prior studies in the published literature that report on motivations of clinicians to publish their investigations of adverse drug reactions that they have observed in clinical practice. Kesselheim et al. reported that whistle-blowers who report on possible fraud by their employers, including four persons who reported that safety data had not been appropriately disseminated, focused on integrity, altruism or public safety, justice, and self-preservation as motivating factors.[13] Formal clinical trial training resulted in one clinician invoking principles of the Belmont Report as part of the impetus for pursuing investigation of potential causes of these serious adverse drug reactions.

Twelve clinicians reported that they had received negative feedback personally or professionally. This too mirrors findings reported for pharmaceutical whistleblowers.[13] Similar experiences have been reported by clinicians who reported rofecoxib-associated cardiac mortality, gadolinium-associated nephrogenic systemic fibrosis, and even aspirin-associated Reye’s syndrome.[16] Kesselheim suggested that for pharmaceutical whistleblowers, ensuring responsible “whistle-blowing” would require interventions that strengthen penalties against retaliation.[11] Similarly, for clinicians, protection against retaliations from pharmaceutical manufacturers are needed. In some instances, this might include financial support if lawsuits against the clinician are filed in an effort to derail the scientific investigation of serious toxicity.

It should be noted that reporting safety findings to the FDA was not a route chosen by 12 clinicians. One clinician who submitted > 60 individual adverse event reports to FDA’s MedWatch program did not receive any feedback from the FDA. While only 1% to 10% of all serious adverse drug reactions are estimated to be reported to the FDA, it is undoubtedly even more rare for a clinician to undertake a detailed investigation of safety data for a large series of patient with the suspected toxicity and then report this information in peer-reviewed medical publications [17]. In one instance, the reporting clinician received a R01 grant from the National Institutes of Health to conduct basic science studies of the toxicity.

In three personal stories we heard, feedback from fellow academics was negative. As suggested by some policy makers, physicians should be able to anonymously report their safety findings to regulatory authorities. This anonymity is not possible with peer-reviewed publications. Extensive influence of pharmaceutical manufacturers on academic communities is concerning, as noted in the high rate of physicians with extensive pharmaceutical funding who participate in FDA Advisory Committee meetings and on medical society-sponsored clinical guideline panels. This influence undoubtedly spills over into academic and FDA environments.

Fourth, personal experience of the clinician interviewees to recommendations for improving pharmaceutical safety are informative. The FDA endorses some of these recommendations with its support of the SENTINEL big-data initiative, which has yielded important safety findings.[18] An important recommendation is for the FDA or the National Institutes of Health to actively support pharmaceutical safety centers of excellence. The Centers for Education and Research of Therapeutics (CERTs) program that was recently disbanded might be a strong candidate for reinstating.[19] Consistent with existing qualitative research studies on barriers to reporting adverse drug reactions, these initiatives do not require additional clinician time or do not result in additional clinician burden.

This study has limitations. We focused on published case series of severe adverse drug reactions that had been investigated by SONAR or RADAR investigators or close collaborators of RADAR or SONAR investigators. These investigators were located at > 50 universities, hospitals, safety organization, or Veterans Administration medical centers. Interview materials obtained from these individuals included highly sensitive personal information that would be difficult to obtain from other clinicians. Our findings therefore may not be generalizable to other clinicians who evaluate serious adverse drug reactions and publish these findings in the peer-reviewed medical literature. Responses to queries about motivation to pursue the scientific investigation may reflect a socially desirable response bias. Finally, recall bias may affect interviewees’ responses.

## Conclusions

Our study provides some of the first reports of clinician motivations and experiences with reporting serious or potentially fatal oncology-associated adverse drug or device reactions. Overall, it appears that negative feedback from pharmaceutical manufacturers and mixed feedback from the academic community and/or the FDA were identified by clinicians who participated in this study. “Big data,” registries, Data Safety Monitoring Boards, and pharmacogenetic studies may facilitate improved pharmacovigilance efforts for oncology-associated adverse drug reactions.[20,21] These initiatives overcome concerns related to complacency, indifference, ignorance, and system-level problems as barriers to documenting and reporting adverse drug events- barriers that have been previously reported for clinician reporting of serious adverse drug reactions. [16,17,19]

## Supporting information

S1 Interview Guide Interview guide for drug researchers. (DOCX)
