# Supplementary material for: Caveat Medicus: Clinician experiences in publishing reports of serious oncology-associated adverse drug reactions
Source: PLoS One. 2019 Jul 31;14(7):e0219521. doi: 10.1371/journal.pone.0219521 (PMC6668902; doi:10.1371/journal.pone.0219521)
Supplement: S1 Interview Guide — (DOCX) [file pone.0219521.s001.docx]

**Supporting information**

S1. Appendix.doc1

**Interview Guide for Drug Researchers and Activist/Informants**

**Legend**

ADR - adverse drug reaction

sADR - severe adverse drug reaction

Sonar - the Southern Network on Adverse Reactions University of South Carolina College of Pharmacy

Radar - Research on Adverse Drug events And Reports (Northwestern University)

pharmacovigilance investigator - someone who investigates the occurrence of ADRs and sADRs as a researcher or an experiencer of negative drug side effects or interactions.

*Please note that drug X is a generic term that will be used to protect the confidentiality of participants and/or their research findings.

**Researcher Introduction**

1. Self
2. Study purpose in brief
3. Data protection protocols

**Background**

1. Please describe your background in the discipline of pharmacovigilance and briefly review experiences in each of the most important adverse drug reactions (Up to three).
2. Please describe your professional background and the settings where you conducted your pharmaceutical safety investigations and reporting efforts?
3. Under what circumstances did you first become familiar with drug X and with its safety concerns?
4. How long had you investigated drug X before safety concerns were noted? Have you or anyone in your family or anyone close to you personally experienced the safety concern that you investigated?
5. What type of financial support did you receive for studying drug X? (Probe, were there other types of benefits, as well?)

**Discovery and Early Problematics**

1. When did you first begin to note a safety with drug X?
2. Under what circumstances did this arise?
3. How did you investigate this problem?
4. How did you initially report your concerns? (Probe: to whom? Were there others to whom you reported the problem?)
5. What type of responses where you met upon reporting the problem?
6. Please describe the circumstances that led you to decide to publicly report that drug X caused a sADR.
7. What happened as a result?
8. Did your funding for the scientific investigation come from a grant? A contract? Did you report the funding to the grant or contract sponsor?

**Short-term actions**

1. What happened after your findings or report of problems with drug X were published or presented? Did you receive coverage in the New York Times, Wall Street Journal, USA Today, local newspaper, local television, NPR, or other places?
2. What were the next events that took place? Did individuals contact you, interview you? Did pharma meet with you? Did lay press meet or interview you? Did your institution meet with you?
3. Can you describe how the events unfolded from that point? Did you experience any lawsuits?

**Follow-up responses to the short-term actions**

1. How would you describe the general response of the FDA, your institution, your medical society, and the manufacturer to the safety report about drug X?
2. How would you describe the general response of the pharmaceutical company?
3. What would you say the main core values that led you to decide to report the safety concern? What were the main drivers of deciding who to report these concerns to?
4. What would you say your guiding ethical principles in this situation were? Did you view the reporting effort as a public health initiative? Medical research? Public policy activity?
5. How large of a role would you say values or ethics play in your decision to move forward in reporting the safety concern with drug X?
6. Did you feel that those ethical principles were maintained after you reported the safety concerns with drug X?
7. Did you feel that integrity of your institution, the manufacturer, the FDA, your academic peers, or yourself was maintained after you reported the safety problem with drug X? Did you feel that your personal reputation and/or integrity had been compromised or enhanced as a result of reporting the safety concern with drug X?
8. To what extent do you believe that ethical behavior of your institution, the manufacturer, the FDA, your academic peers, and others was maintained after your findings?
9. Can you please describe any effects on your own personal health of reporting drug X?
10. Can you please describe any legal ramifications?
11. Can you please describe any impact on your career?
12. Can you please describe any personal financial ramifications?
13. To what extent was dignity and respect for you maintained by the pharmaceutical company?
14. To what extent were your personal and professional contacts supportive or unsupportive of you after your experience with reporting safety concerns with drug X?
15. What do you feel your duty or ethical responsibility was with respect to reporting a safety concern with drug X?
16. To what extent was good intention toward you displayed by pharma?
17. Did you sense any ethical transgressions before, during, and/or after reporting safety concerns with drug X?
18. Can you explain how those played out?
19. Do you believe this situation could have been handled more ethically- before the report was produced; during the time it was being disseminated, and/or after the dissemination occurred?
20. Did you feel that your integrity as a researcher and dignity as a person was challenged or/even compromised?
21. Looking back, do you have any thoughts on different strategies, policies, or other actions that would improve pharmaceutical safety reporting efforts by you going forward?
22. Broadly thinking, are there any strategies, initiatives, or other avenues that might improve the chances of having a positive experience with reporting an adverse drug reaction for any other researcher (based on lessons learned from your experience)?

**Outcomes**

- - - 1. What kinds of impact has reporting the sADR had on your life? How so?
      2. What types of ongoing legal constraints and actions were taken? What kind of actions were taken by your institution? By your funder? By the pharmaceutical manufacturer? By your co-researchers at your institution or in your medical society?
      3. Can you explain the impact of any litigation on your person, career, and finances? (Probe: Are there examples you can share?)
      4. Were there other outcomes – personal or professional? How were these positive or negative?
      5. To what extent, if any, did you feel positive results- such as increased reputation, improved access with the FDA, and other events?
      6. Did you feel disrespected? Degraded? Threatened? At risk for harm?

**Contingency Questions: Activists Only**

1. What kinds of activities did you engage and to widen knowledge about the sADR of drug X?
2. What types of lobbying or legislative initiatives were attempted? Enacted?
3. What types of media relations activities were attempted?
4. What types of communication with the FDA was attempted?
5. What lines of communication did you attempt to use that were unsuccessful?
6. Can you explain the impact of any litigation or new regulations on the issue?
7. Were there other outcomes – personal or professional? How were these positive or negative?
8. To what extent, if any, did you feel disrespected? Degraded? Threatened? Harmed?
9. To what extent, if any, did you feel successful at changing the issue? Regulations?
10. Were there any other activist activities in which you engaged directed at this particular drug or pharmaceutical company?
11. Who do you think should have held that responsibility?
12. Who exceeded your expectations or failed to uphold their ethical responsibility?
13. To what extent do you believe the clinical trial results were reported honestly?
14. Do you believe that the clinical trial results were thorough enough?

**Future**

1. How do you think your experience might encourage pharmaceutical company employees to consider ethics in their activities?
2. What means of improvement could there be on the pharmaceutical side of considering problematics with a drug like X?
3. What can or should be done to encourage the reporting of drug interactions and problems?
4. How can disincentives or barriers that you faced be lessened for future reporting of sADRs?
5. Do you feel your reporting about drug X was worthwhile? Would you have done it again?
6. What steps do you think could be taken to help encourage future generations to report problems with a drug? (Probe, are there incentives for protections that could be put in place, etc.?)
7. What ethical standards do you think should be emphasized in a situation such as yours?
8. Who should take responsibility for the negative outcomes you have experienced?
9. What guidance can you offer it for the right way to handle drug problems with ethics, and dignity, and respect for those who experience sADRs?
10. What future standards would you like to see put in place to help researchers such as yourself when problematics with the drug are encountered?
11. Are there different actions the FDA or pharmaceutical companies could take to encourage drug safety and efficacy? What would you suggest?
12. Is there a question that I should have asked you - or anything else you’d like to tell me that may help in this study?
13. Would you be comfortable examining a transcript of our session to make certain we have gotten all of the details correct? You can elaborate further or correct items at that time.

**Informants only:**

1. Are you comfortable being identified by your first and last name and academic affiliation in publications resulting from this study? ___No ___ Yes; Please list any exclusions: ______________________________________________________________________
2. Are you comfortable being identified by the drug name and interaction in publications resulting from this study (not your personal name)? ___No ___ Yes; Please list any exclusions:_______________________________________________________________

Signature_________________________________ Date: _________________

**All:**

1. Timeline for member check of data.
2. Any debrief necessary.

On behalf of our research team, I’d like to thank you for participating in this study. If you have any additional thoughts, comments, ideas, or questions, please feel free to contact me by phone or email (sbowen@sc.edu). We encourage your continued involvement and comment in our research.
